# Molecular basis and evolutionary cost of a novel macrolides/lincosamides resistance phenotype in *Staphylococcus haemolyticus*

**DOI:** 10.1128/spectrum.00441-23

**Published:** 2023-09-19

**Authors:** Ruilin Xu, Qiang Wang, Shuhua Wu, Hongqiu Wang, Tianqiang Song, Chenhao Zhao, Min Wang, Hong Du, Haifang Zhang

**Affiliations:** 1 The State Key Laboratory of Pharmaceutical Biotechnology, School of Life, Nanjing University, Nanjing, Jiangsu, China; 2 Center for Life Sciences, Academy for Advanced Interdisciplinary Studies, Peking University, Beijing, China; 3 Department of Geriatrics, The Second Affiliated Hospital of Soochow University, Suzhou, Jiangsu, China; 4 Department of General Practice, The Second Affiliated Hospital of Soochow University, Suzhou, Jiangsu, China; 5 Department of Clinical Laboratory, The Second Affiliated Hospital of Soochow University, Suzhou, Jiangsu, China; University of Pittsburgh School of Medicine, Pittsburgh, Pennsylvania, USA

**Keywords:** *Staphylococcus haemolyticus*, novel antibiotic-resistance phenotype, constitutive MLS resistance, *ermC *gene

## Abstract

*Staphylococcus haemolyticus* (*S. haemolyticus*) is a coagulase-negative *Staphylococcus* that has become one of the primary causes of nosocomial infection. After a long period of antibiotic use, *S. haemolyticus* has developed multiple resistance phenotypes for macrolides and lincosamides. Herein, we evaluated four *S*. *haemolyticus* clinical isolates, of which three had antibiotic resistance patterns reported previously. The fourth isolate was resistant to both erythromycin and clindamycin in the absence of erythromycin induction. This novel phenotype, known as constitutive macrolides-lincosamides-streptogramins resistance, has been reported in other bacteria but has not been previously reported in *S. haemolyticus*. Investigation of the isolate demonstrated a deletion in the methyltransferase gene *ermC,* upstream leader peptide. This deletion resulted in constitutive MLS resistance based on whole-genome sequencing and experimental verification. Continuous expression of *ermC* was shown to inhibit the growth of *S. haemolyticus*, which turned out to be the fitness cost with no MLS pressure. In summary, this study is the first to report constitutive MLS resistance in *S. haemolyticus*, which provides a better understanding of MLS resistance in clinical medicine.

**Importance:**

This study identified a novel phenotype of macrolides/lincosamides resistance in *Staphylococcus haemolyticus* which improved a better guidance for clinical treatment. It also clarified the mechanistic basis for this form of antibiotic resistance that supplemented the drug resistance mechanism of *Staphylococcus*. In addition, this study elaborated on a possibility that continuous expression of some resistance genes was shown to inhibit the growth of bacteria themselves, which turned out to be the fitness cost in the absence of antibiotic pressure.

## INTRODUCTION

*Staphylococcus* is a genus of Gram-positive bacteria that is widely distributed in nature and can be divided into two categories, coagulase-positive and -negative. The former category primarily comprises *Staphylococcus aureus*, while the latter category includes *Staphylococcus epidermidis* and *S. haemolyticus*. Coagulase-negative *Staphylococcus* (CoNS) has long been considered a harmless microbe (1). Since the 1980s, reports of opportunistic infections caused by CoNS have gradually increased (2 – 4). *S. haemolyticus*, which produces hemolysins and enterotoxins (5), is particularly pathogenic for ICU patients (6).

*S. haemolyticus* had multiple antibiotic resistance phenotypes: resistance to erythromycin or clindamycin and resistance to erythromycin and clindamycin with erythromycin induction. The latter is known as an inducible macrolides-lincosamides-streptogramins (MLS) phenotype (7). *S. haemolyticus* has three primary antibiotic resistance mechanisms: (i) pumping antibiotics out by membrane transporter proteins that are encoded by an ATP binding cassette (ABC) transport superfamily, including *vga(A)_LC_*, and *msrA* (8, 9); (ii) inactivation of antibiotics, such as by *mphC*, which encodes a macrolide phosphotransferase that modifies antibiotics by phosphorylation (10); and (iii) an alteration of the antibiotic’s target, preventing the antibiotic from binding to its target. For example, the ribosomal RNA methyltransferase *Erm* family adds a methyl modification to bacterial 23S rRNA, which renders the antibiotic unable to bind to the 50S ribosomal subunit (11).

With excessive use of antibiotics, many bacteria have developed drug resistance. These emerging antibiotic resistance phenotypes have become a serious clinical issue that has resulted in fatality for an increasing number of patients. Herein, we explored emerging bacterial phenotypes that may be capable of reducing the spread of drug-resistant pathogens. The results of this study provided a better understanding of drug resistance and improved guidance for clinical treatment.

## MATERIALS AND METHODS

### Bacterial strains and media

*S. haemolyticus* strains A, B, C, and D used in this study were initially isolated from patients with invasive clinical infections at the Second Hospital of Soochow University (Jiangsu, China). Plasmid pBT2 (*Staphylococcus-Escherichia* shuttle vector) was used as a backbone (12). Since pBT2 is a temperature-sensitive plasmid, it can be lost when incubated at higher temperatures. Therefore, all strains with pBT2-related plasmid were cultured at 30°C.

### Genome sequencing and assembly

We performed next-generation sequencing (Illumina, Hiseq2500, PE100) of these four isolates of *S. haemolyticus*. Raw reads were trimmed by FastQC (13), KAT (14), and BBTools (15). To achieve better assembly results, we integrated five tools to assemble clean reads: SuperReads from Masurca (16), BCALM 2 (17), Tadpole from BBTools, SPAdes (18), and Megahit (19) (Fig. S1). Assemblies of these tools were imported into Sequencher (Gene Codes, MI, USA), and the consensus sequences were manually verified and selected to form final assembly files according to the coverage depth of reads. BUSCO (20) was used to evaluate the accuracy and completeness of the final assembly results. The raw sequencing data and final assembly files were uploaded to NCBI database (accession numbers SRR14777770-SRR14777773).

### Antibiotic resistance gene analysis

Prokka was used to annotate the assembled sequences (21). The potential resistance genes were analyzed by Resistance Gene Identifier (RGI) (22).

### Sequencing reads depth calculation

We aligned sequencing reads to genome assembly files with the Burrows-Wheeler-Alignment tool (23) and then used Mosdepth (24) to calculate the read depth from two levels --contigs and genes.

### Plasmid construction and transformation

The *ermC* gene was amplified from the genomic DNA of *S. haemolyticus* strain C and inserted into the pBT2 backbone to form pBT2-*ermC*. The upstream leader peptide sequence (short for LP) of *ermC* was obtained from the *S. haemolyticus* strain PK-01 chromosome (NCBI Accession: CP035541, region: 2,573,055–2,573,114) and synthesized by the GenScript Biotech Co. Plasmids were transformed into *S. aureus* strain ATCC25923 by electro-transformation at 2.0 kV, 25 µF, and 100 Ω. All primers for plasmid construction were provided in Table S2.

### Isolation of RNA and RT-PCR

Total RNA was extracted by the TRIzol method (Invitrogen, Carlsbad, CA, USA). RNA purity and integrity were examined by RNA electrophoresis in 1.0% (wt/vol) agarose gel. One microgram of RNA was treated with DNase I prior to cDNA synthesis using a Revert-Aid First Strand cDNA Synthesis Kit (Thermo Scientific, MA, USA). RT-PCR was performed with specific primers for *ermC* and *Staphylococcus* 16S rRNA. All RT-PCR primers were provided in Table S2.

### Antimicrobial susceptibility test

The turbidity of *S. aureus* transformed with recombinant plasmids was adjusted to a 0.5 McFarland turbidity (1.5 × 10^8^ CFU/mL) and then evenly coated onto Mueller-Hinton agar medium. After 2 min, an erythromycin susceptibility test disc (15 µg) and a clindamycin susceptibility test disc (2 µg) were placed onto the middle of the plate. The distance between the disc and the edge of the Petri dish was more than 15 mm, while the two discs were 15–26 mm apart. The plate was incubated at 30°C for 20–24 h before the results were assessed. The current CLSI M100 (Performance Standards for Antimicrobial Susceptibility Testing) was used to determine whether the bacteria were resistant to the antibiotics.

### Growth curve measurement

*S. aureus* transformed with different plasmids was cultured as above (30°C, trypticase soy broth, chloramphenicol, 200 rpm/min shaker). Bacterial count was measured every 2 h for 20 h.

## RESULTS

### Identification of a novel phenotype for macrolides/lincosamides resistance in *S. haemolyticus*

In the Second Hospital of Soochow University, we isolated four strains of *S. haemolyticus*, identified as A, B, C, and D. During routine drug sensitivity testing, strains A and B were resistant to clindamycin and sensitive to erythromycin. Strain D was resistant to erythromycin and sensitive to clindamycin. Strain C was resistant to both erythromycin and clindamycin. And it could tolerate clindamycin directly without erythromycin induction (constitutive MLS resistance). These phenotypes for strains A, B, and D are common in clinical practice, and their mechanisms of drug resistance have been previously described. However, the phenotype of strain C has not been reported previously.

Constitutive MLS resistance has been reported for *S. aureus* (25, 26), *S. epidermidis* (27), *Streptococcus pyogenes* (28), *Streptococcus pneumoniae,* and *Enterococcus faecalis* (29). However, no similar phenotype has been reported for *S. haemolyticus*. Herein, the basis for this antibiotic resistance was examined.

### Genome assembly and antibiotic resistance gene analysis

**Fig 1:**
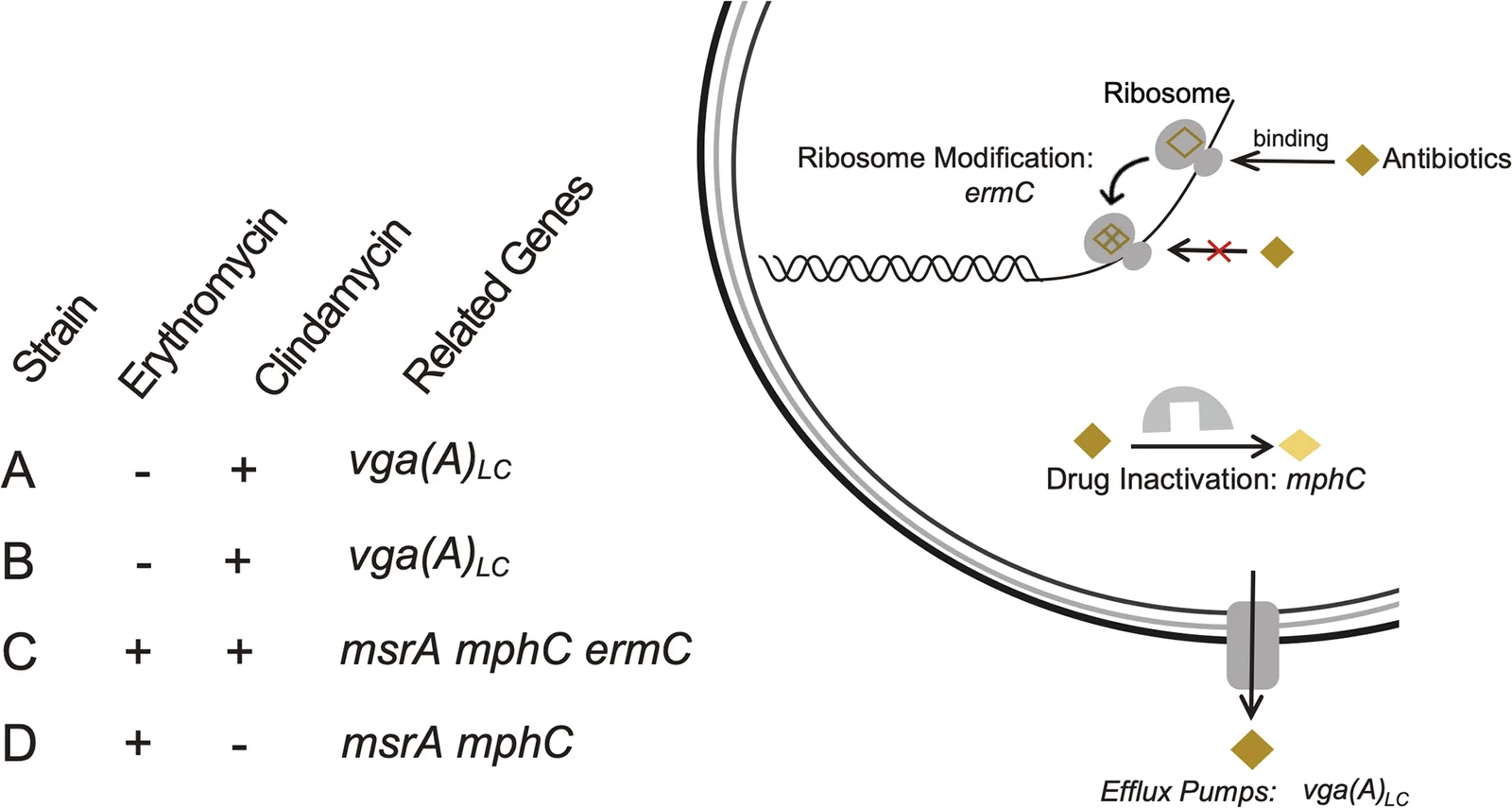
Resistance genes related to erythromycin and clindamycin were detected in four *S*. *haemolyticus* strains A, B, C, and D. Plus or minus signs represent strains resistant or sensitive to antibiotics, respectively. Resistance mechanisms for these genes were shown on the right. Diamonds represent various antibiotics.

**Fig 2:**
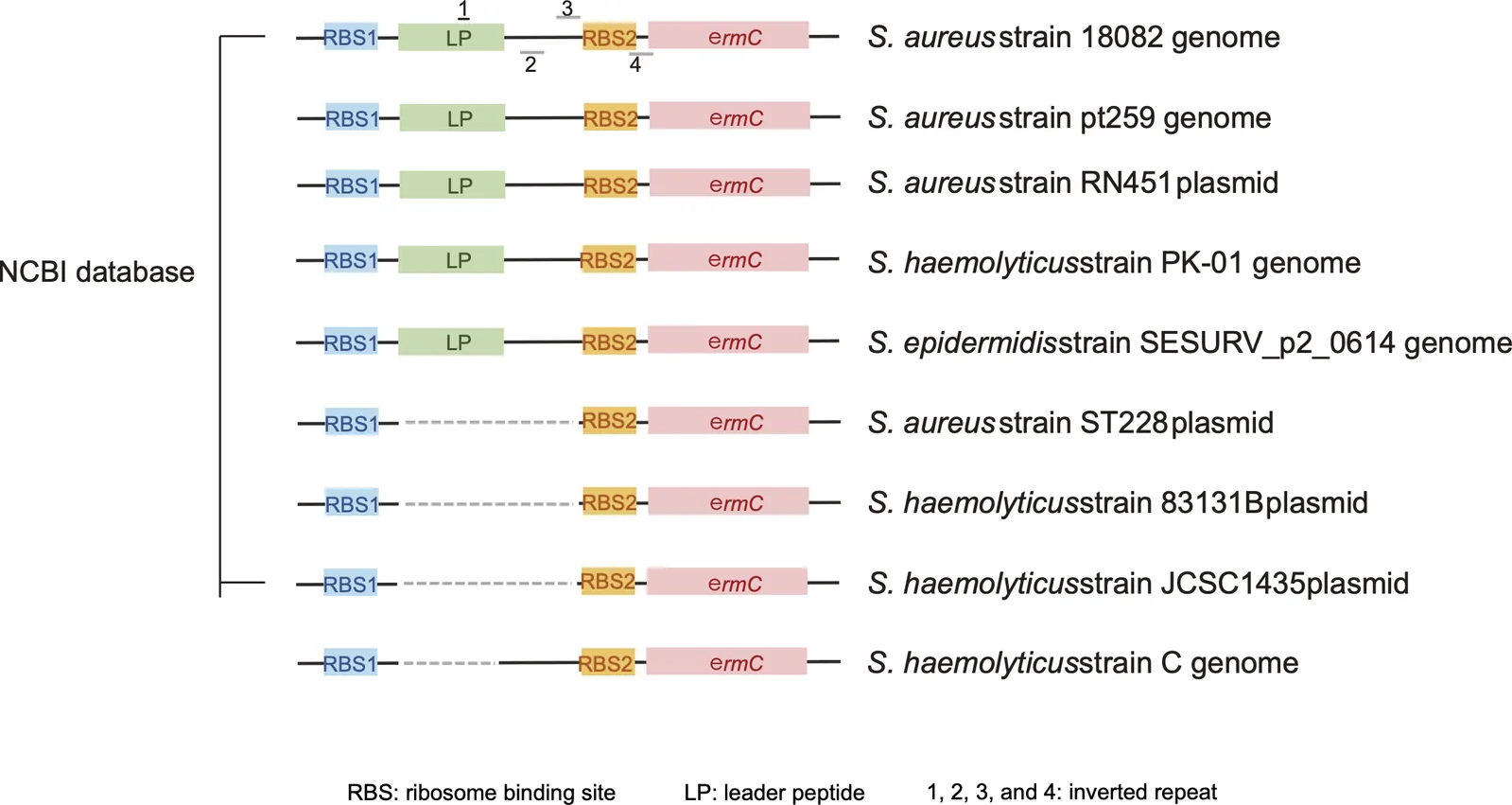
Identification of the target gene, *ermC*. Comparison of the *ermC* sequence identified from strain C with its homolog in NCBI. A short leader peptide was missing upstream.

**Fig 3:**
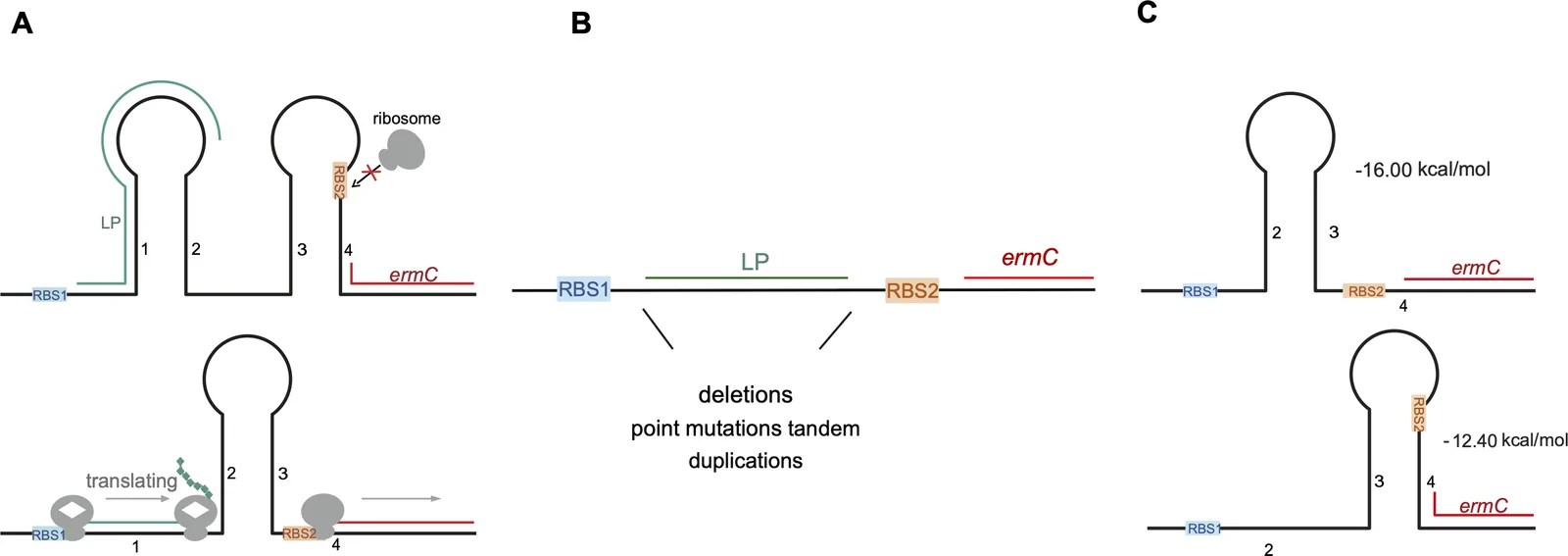
Mechanism of inducible (**A**) and constitutive (**B**) MLS resistance. (**C**) Secondary structure prediction for a region upstream of *ermC* in *S. haemolyticus* strain C. (**A**) *ermC* mRNA was in an inactive conformation (upper portion of the panel) due to the structure of its 5*ʹ* end, which had a set of four inverted repeats paired as hairpin structure 1:2 and 3:4 that stalled the ribosomes and sequestered the initiation sequences for *ermC*. Thus, the methylase could not be synthesized because the initiation motifs for translation were not accessible to ribosomes. In the presence of inducer macrolides, the antibiotic (white diamond) first bound to the ribosome, dramatically slowing down the ribosome’s speed and enabling translation of the leader peptide upstream of *ermC*. This induced the destabilization of the two hairpin structures and favored the association between inverted repeats 2 and 3 to form a new hairpin structure 2:3, which uncovered the initiation sequences that increase the translation efficiency of *ermC*. (**B**) The variations in the leader peptide decreased the stability of the hairpin structure and render *ermC* available for translation, resulting in constitutive MLS resistance. (**C**) In *S. haemolyticus* strain C, the deletion of the leader peptide caused the other inverted repeats to pair as hairpin structure 2:3 or 3:4. The minimum free energy of hairpin structure 2:3 was lower than 3:4, indicating that the secondary structure upstream of *ermC* was more likely to form as hairpin structure 2:3, which would expose initiation sequences for translation.

The genomes of the four *S*. *haemolyticus* clinical isolates were sequenced and assembled. The assembly results are listed in Table 1, with all four assemblies demonstrated to be of high quality and high coverage. Potential resistance genes were analyzed by RGI (Table S1). Based on previous studies, several genes were found to be related to erythromycin and clindamycin resistance, *vga(A)_LC_*, *msrA*, *mphC,* and *ermC*. As expected, antibiotic resistance genes found in strains A, B, and D corresponded to their respective phenotypes (Fig. 1). Since the phenotype of strain C was unique, we focused analysis on that strain. By comparison of the resistance genes of strain C with their homologs in NCBI, we found that a short leader peptide was missing upstream of *ermC* (Fig. 2). This leader peptide was found to be essential for *ermC* expression during erythromycin induction. Ribosome binding by erythromycin permitted translation of the leader peptide, which rearranged hairpin structures composed of four inverted repeats and exposed the initiation motifs of *ermC* for translation (Fig. 3A) (30, 31). While in *S. aureus*, the variation of the leader peptide rendered the downstream *ermC* gene to switch from inducible expression to constitutive expression, resulting in constitutive MLS resistance (Fig. 3B) (32, 33). Deletion of the leader peptide resulted in inverted repeat 1, allowing the others to pair as hairpin structures 2:3 or 3:4. Through RNAfold WebServer, we found that the secondary structure upstream of *ermC* was more likely to form as hairpin structure 2:3, which would expose an initiation sequence for the methylase translation (Fig. 3C). As such, we hypothesized that constitutive MLS resistance in *S. haemolyticus* strain C was due to deletion of the leader peptide upstream of *ermC*.

**TABLE 1:** Genome assembly results for the four *S. haemolyticus* strains

Strain	Reads number	N50	Contigs	Total length	BUSCO complete (%)
A	12,932,836	97,887	41	2,572,850	100
B	13,832,808	84,894	47	2,426,878	100
C	8,770,750	66,588	63	2,652,164	99.2
D	13,103,638	90,527	52	2,596,861	100

### The *ermC* gene found in *S. haemolyticus* strain C was located on a plasmid

**Fig 4:**
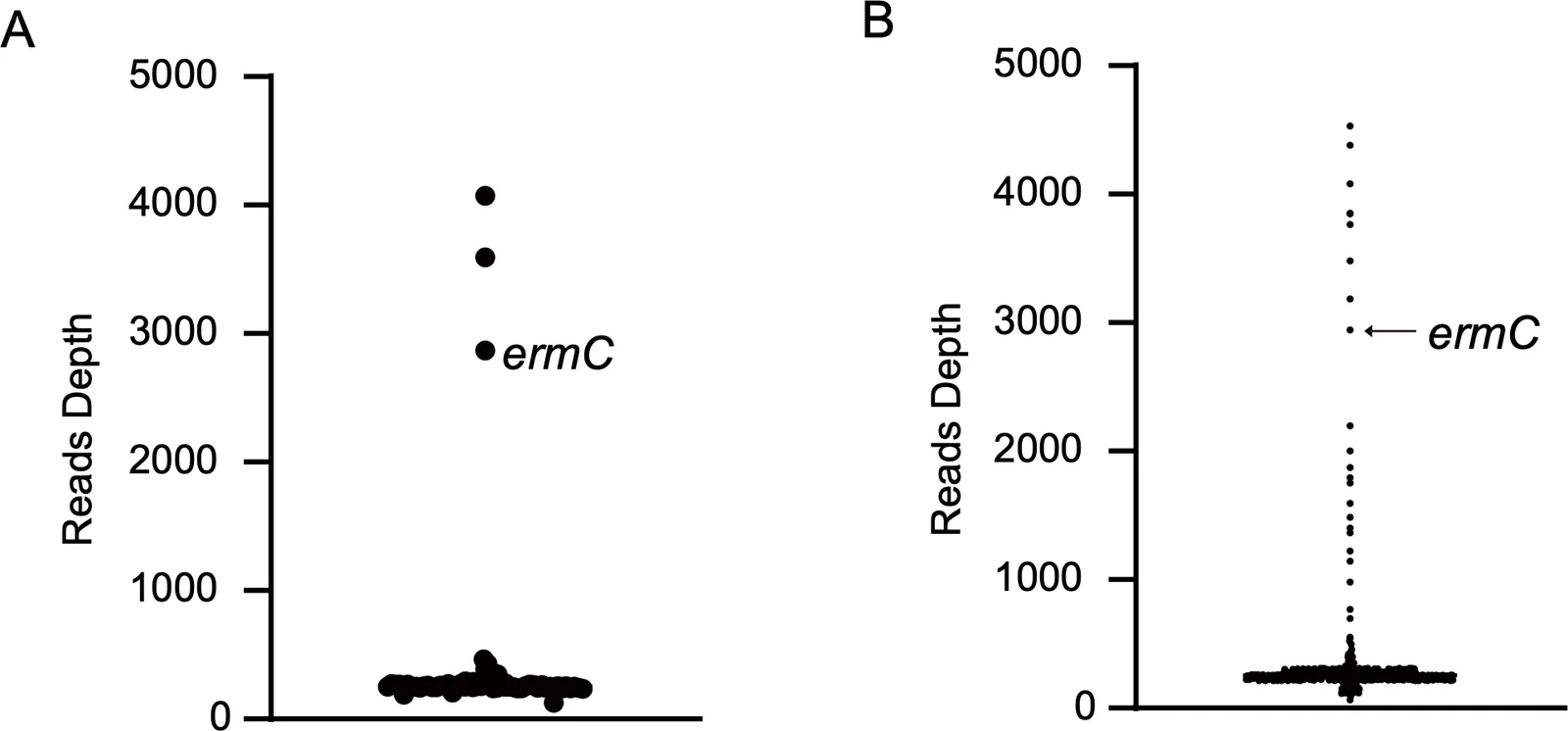
Read depth of *S. haemolyticus* strain C. (**A**) Black dots represented contigs from genome assembly files. The read depth of most contigs was consistent, while several were much higher than others, such as the one containing *ermC*. (**B**) Small black dots represented each annotated gene, with the read depth of *ermC* far above the average.

We aligned raw sequencing data with plasmid-specific elements in *Staphylococcus* spp. and found the specific elements in sequencing reads, indicating that genome sequencing results contained bacterial plasmid reads. We performed read depth calculation for *S. haemolyticus* strain C genome assembly files (the read depth calculation results for *S. haemolyticus* strains A, B, and D are shown in Fig. S2). Results (Fig. 4) showed read depth for both the contig containing *ermC* or the *ermC* gene itself were significantly higher, suggesting that *ermC* was most likely located on a plasmid. Based on *in silico* studies, we performed experimental validation and obtained the complete plasmid which closely resembled other known drug-resistant plasmids reported in *S. aureus*.

### Deletion of the leader peptide upstream of *ermC* resulted in MLS constitutive resistance of *S. haemolyticus*

To validate the hypothesis that the constitutive MLS resistance of *S. haemolyticus* strain C was caused by deletion of the leader peptide upstream of *ermC*, we amplified the intact *ermC* gene from strain C and constructed a recombinant plasmid, pBT2-*ermC*. A synthesized upstream leader peptide (short for LP) sequence was inserted upstream of *ermC* in plasmid pBT2-*ermC* to form pBT2-LP-*ermC*. Due to the absence of a widely used standard strain of *S. haemolyticus* for drug sensitivity tests, we chose *S. aureus* for analysis. *S. aureus* ATCC25923 is usually used as a negative control strain for antimicrobial susceptibility testing (34).

**Fig 5:**
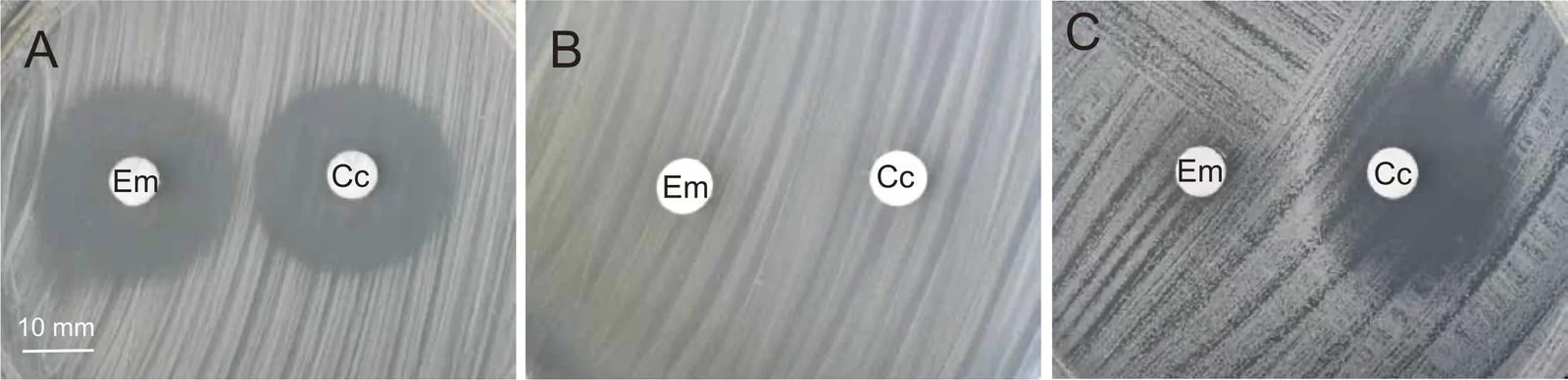
Results of antimicrobial susceptibility testing of *S. aureus* ATCC25923. Em, erythromycin; Cc, clindamycin. (**A**) *S. aureus* carrying backbone plasmid, pBT2, was sensitive to both erythromycin and clindamycin. (**B**) *S. aureus* carrying pBT2-*ermC* showed constitutive MLS resistance. (**C**) *S. aureus* carrying pBT2-LP-*ermC* showed clindamycin resistance only on the disc side close to the erythromycin susceptibility test disc, resulting in inducible MLS resistance.

We performed a double disk diffusion test to determine MLS inducible/constitutive resistance (Fig. 5). *S. aureus* carrying pBT2-*ermC* was unaffected by erythromycin or clindamycin, forming a phenotype of constitutive MLS resistance. Whereas the strain harboring pBT2-LP-*ermC* showed clindamycin resistance only on the side of the disc close to the erythromycin susceptibility test disc (inhibition zone radius ≈ 7 mm). The side of the disc away from the erythromycin disc remained clindamycin sensitive (inhibition zone radius ≈ 15 mm) resulting in the formation of a D-Circle, which indicated that erythromycin induction was required for resistance to clindamycin. Therefore, constitutive MLS resistance by *S. haemolyticus* strain C was due to leader peptide deletion upstream of *ermC*.

### The 23S rRNA methylation, modified by *ermC,* may have adverse effects on bacterial growth

**Fig 6:**
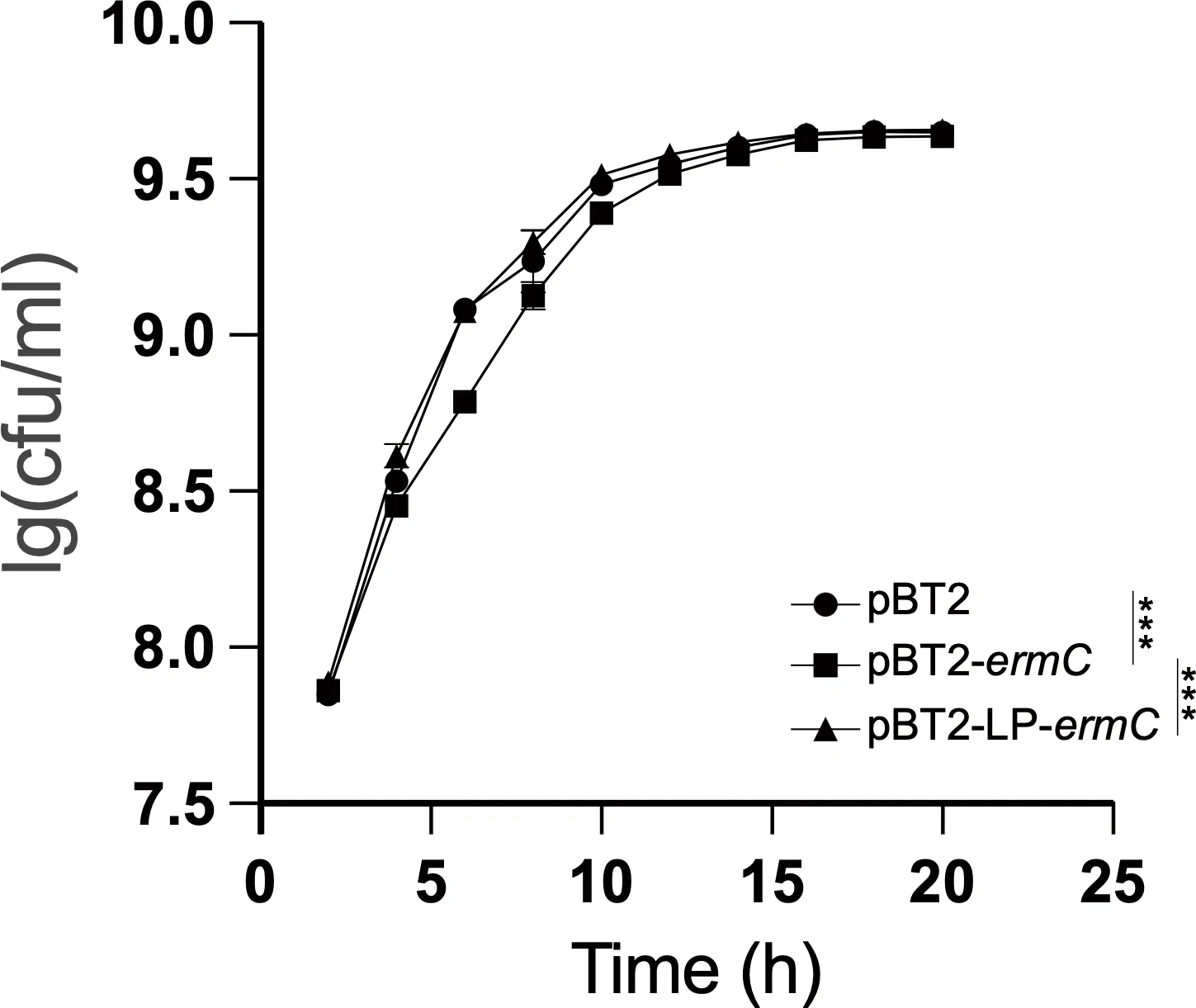
Growth curves of *S. aureus* ATCC25923 transformed with different plasmids. ANOVA was used for statistical analysis, ***P < 0.001.

Multiple studies have demonstrated constitutive MLS resistance phenotypes for common pathogenic *Staphylococcus* strains (35, 36). Then since bacteria could constitutively resist antibiotics, why have they evolved inducible resistance. An interesting observation in this study suggested a possibility. After different plasmids were transformed into *S. aureus*, growth rates were found to vary considerably. To confirm these results, we performed a growth curve analysis, as shown in Fig. 6. Results showed that the growth of *S. aureus* carrying pBT2-*ermC* was significantly slower than the others. We speculated that this observation might be due to the adverse effects of *ermC*-mediated 23S rRNA methylation. Compared with unmethylated ribosomes, the translation speed of methylated ribosomes decreased, directly affecting bacterial metabolism and survival, while imposing an enormous fitness cost in the absence of antibiotics.

## DISCUSSION

In this study, we analyzed four *S*. *haemolyticus* strains isolated from clinical patients. One strain exhibited a surprising drug-resistant phenotype, constitutive MLS resistance. Although constitutive MLS resistance has been reported in *Staphylococcus* spp., *Streptococcus* spp*.,* and *Enterococcus* spp*.,* this was a novel observation for *S. haemolyticus*. We found strain C contained a methyltransferase gene, *ermC,* with a deletion in the upstream leader peptide, which resulted in constitutive MLS resistance. In this manner we identified, for the first time, a variation in the *ermC* gene of *S. haemolyticus*. The plasmids that mimicked the wild-type and varied genotype were constructed and transformed into *S. aureus* for susceptibility testing. Based on mRNA expression and drug resistance, it was clear that this small leader peptide was critical to the regulation of MLS resistance.

Constitutive MLS resistance by clinical pathogenic bacteria has become an increasingly important medical issue due to the selective pressure of antibiotic use. Based on the principle of fitness cost, restricting antibiotic usage could be beneficial for the elimination of constitutive drug-resistant bacteria. Such bacteria expressed nonessential genes that might aggravate extra strain burden, competing with other strains when antibiotic selection pressure decreased or disappeared.

It has been reported that multidrug resistance was exhibited by 75% of *S. haemolyticus* clinical isolates (37). Intraspecies transfer of staphylococcal cassette chromosome *mec* (SCC*mec*) indicated that *S. haemolyticus* could be a reservoir of resistance genes (38, 39). The genome plasticity of *S. haemolyticus* is characterized by many insertion sequences and SNPs, contributing to the acquisition of antibiotic resistance (40). Various *Staphylococcus* spp*.,* including *S. haemolyticus,* may constitute a vast reservoir of resistance genes as well as multi-resistant strains, with frequent intraspecific or interspecies gene transfer, which shortens the usefulness of antibiotics.

Our study has identified a novel phenotype of macrolides/lincosamides resistance in *S. haemolyticus*. However, this resistance phenotype and corresponding mechanism have been found in *S. aureus* (32, 33), so our discovery was not the first in *Staphylococcus* spp. In addition, we found that sustained expression of *ermC* may affect bacterial metabolism and survival, and we have provided some explanations for this phenomenon. But we have not proven our hypothesis through actual biological experiments. This led to our viewpoint that bacteria with inducible resistance had a competitive advantage in survival compared to bacteria with constitutive resistance. This point needs to be proven through more experiments in the future.

### Conclusions

In summary, this study identified a novel phenotype of macrolides/lincosamides resistance in *Staphylococcus haemolyticus* and clarified the mechanistic basis for this form of antibiotic resistance. Furthermore, we demonstrated the expression of the 23S rRNA methylase gene, *ermC,* to attenuate the metabolism and growth of *S. haemolyticus*, suggesting it could be a fitness cost in the absence of antibiotic pressure.

## Data Availability

The bacterial raw sequencing data and final assembly files were uploaded to the NCBI database (accession numbers SRR14777770-SRR14777773).

## SUPPLEMENTAL MATERIAL

Figures S1 and S2 spectrum.00441-23-s0001.pdf Schematic diagram of merge contigs in genome assembly process.

Figure S3 spectrum.00441-23-s0002.pdf Identification of *ermC* expression in *S. aureus* ATCC25923 transformed with different plasmids.

Tables S1 and S2 spectrum.00441-23-s0003.pdf All potential resistance genes identified in *S. haemolyticus* strains ABCD; primers used in this study.

Supplemental material information spectrum.00441-23-s0004.pdf Legends for supplemental figures and tables.

ASM does not own the copyrights to Supplemental Material that may be linked to, or accessed through, an article. The authors have granted ASM a non-exclusive, world-wide license to publish the Supplemental Material files. Please contact the corresponding author directly for reuse.

## References

[1] Natsis NE , Cohen PR . 2018. Coagulase-negative Staphylococcus skin and soft tissue infections. Am J Clin Dermatol 19:671–677. doi:10.1007/s40257-018-0362-9 29882122

[2] Tabe Y , Nakamura A , Oguri T , Igari J . 1998. Molecular characterization of epidemic multiresistant Staphylococcus haemolyticus isolates. Diagn Microbiol Infect Dis 32:177–183. doi:10.1016/s0732-8893(98)00118-7 9884833

[3] Huebner J , Goldmann D . 1999. Coagulase-negative staphylococci. Annu Rev Med 50:223–236. doi:10.1146/annurev.med.50.1.223 10073274

[4] Arciola CR , Campoccia D , An YH , Baldassarri L , Pirini V , Donati ME , Pegreffi F , Montanaro L . 2006. Prevalence and antibiotic resistance of 15 minor staphylococcal species colonizing orthopedic implants. Int J Artif Organs 29:395–401. doi:10.1177/039139880602900409 16705608

[5] Valle J , Gomez-Lucia E , Piriz S , Goyache J , Orden JA , Vadillo S . 1990. Enterotoxin production by staphylococci isolated from healthy goats. Appl Environ Microbiol 56:1323–1326. doi:10.1128/aem.56.5.1323-1326.1990 2339886 PMC184403

[6] Piette A , Verschraegen G . 2009. Role of coagulase-negative staphylococci in human disease. Vet Microbiol 134:45–54. doi:10.1016/j.vetmic.2008.09.009 18986783

[7] Levin TP , Suh B , Axelrod P , Truant AL , Fekete T . 2005. Potential clindamycin resistance in clindamycin-susceptible, erythromycin-resistant Staphylococcus aureus: report of a clinical failure. Antimicrob Agents Chemother 49:1222–1224. doi:10.1128/AAC.49.3.1222-1224.2005 15728934 PMC549252

[8] Novotna G , Janata J . 2006. A new evolutionary variant of the streptogramin a resistance protein, Vga(A)LC, from Staphylococcus haemolyticus with shifted substrate specificity towards lincosamides. Antimicrob Agents Chemother 50:4070–4076. doi:10.1128/AAC.00799-06 17015629 PMC1693986

[9] Ross JI , Eady EA , Cove JH , Cunliffe WJ , Baumberg S , Wootton JC . 1990. Inducible erythromycin resistance in staphylococci is encoded by a member of the ATP-binding transport super-gene family. Mol Microbiol 4:1207–1214. doi:10.1111/j.1365-2958.1990.tb00696.x 2233255

[10] Lüthje P , Schwarz S . 2006. Antimicrobial resistance of coagulase-negative staphylococci from bovine subclinical mastitis with particular reference to macrolide-lincosamide resistance phenotypes and genotypes. J Antimicrob Chemother 57:966–969. doi:10.1093/jac/dkl061 16524893

[11] Poehlsgaard J , Douthwaite S . 2005. The bacterial ribosome as a target for antibiotics. Nat Rev Microbiol 3:870–881. doi:10.1038/nrmicro1265 16261170

[12] Brückner R . 1997. Gene replacement in Staphylococcus carnosus and Staphylococcus xylosus. FEMS Microbiol Lett 151:1–8. doi:10.1111/j.1574-6968.1997.tb10387.x 9198277

[13] Wingett SW , Andrews S . 2018. FastQ screen: a tool for multi-genome mapping and quality control. F1000Res 7:1338. doi:10.12688/f1000research.15931.2 30254741 PMC6124377

[14] Mapleson D , Garcia Accinelli G , Kettleborough G , Wright J , Clavijo BJ . 2017. KAT: a K-mer analysis toolkit to quality control NGS datasets and genome assemblies. Bioinformatics 33:574–576. doi:10.1093/bioinformatics/btw663 27797770 PMC5408915

[15] Bushnell B . 2014. BBMap: a fast, accurate, splice-aware aligner. Lawrence Berkeley National Laboratory

[16] Zimin AV , Marçais G , Puiu D , Roberts M , Salzberg SL , Yorke JA . 2013. The MaSuRCA genome assembler. Bioinformatics 29:2669–2677. doi:10.1093/bioinformatics/btt476 23990416 PMC3799473

[17] Chikhi R , Limasset A , Medvedev P . 2016. Compacting de Bruijn graphs from sequencing data quickly and in low memory. Bioinformatics 32:i201–i208. doi:10.1093/bioinformatics/btw279 27307618 PMC4908363

[18] Bankevich A , Nurk S , Antipov D , Gurevich AA , Dvorkin M , Kulikov AS , Lesin VM , Nikolenko SI , Pham S , Prjibelski AD , Pyshkin AV , Sirotkin AV , Vyahhi N , Tesler G , Alekseyev MA , Pevzner PA . 2012. Spades: a new genome assembly algorithm and its applications to single-cell sequencing. J Comput Biol 19:455–477. doi:10.1089/cmb.2012.0021 22506599 PMC3342519

[19] Li D , Liu C-M , Luo R , Sadakane K , Lam T-W . 2015. MEGAHIT: an ultra-fast single-node solution for large and complex metagenomics assembly via succinct de Bruijn graph. Bioinformatics 31:1674–1676. doi:10.1093/bioinformatics/btv033 25609793

[20] Simão FA , Waterhouse RM , Ioannidis P , Kriventseva EV , Zdobnov EM . 2015. BUSCO: assessing genome assembly and annotation completeness with single-copy orthologs. Bioinformatics 31:3210–3212. doi:10.1093/bioinformatics/btv351 26059717

[21] Seemann T . 2014. Prokka: rapid prokaryotic genome annotation. Bioinformatics 30:2068–2069. doi:10.1093/bioinformatics/btu153 24642063

[22] Jia B , Raphenya AR , Alcock B , Waglechner N , Guo P , Tsang KK , Lago BA , Dave BM , Pereira S , Sharma AN , Doshi S , Courtot M , Lo R , Williams LE , Frye JG , Elsayegh T , Sardar D , Westman EL , Pawlowski AC , Johnson TA , Brinkman FSL , Wright GD , McArthur AG . 2017. CARD 2017: expansion and model-centric curation of the comprehensive antibiotic resistance database. Nucleic Acids Res 45:D566–D573. doi:10.1093/nar/gkw1004 27789705 PMC5210516

[23] Li H . 2013. Aligning sequence reads, clone sequences and assembly contigs with BWA-MEM. ArXiv 1303. doi:10.48550/arXiv.1303.3997

[24] Pedersen BS , Quinlan AR . 2017. Mosdepth: quick coverage calculation for genomes and exomes. Bioinformatics. doi:10.1101/185843 PMC6030888 29096012

[25] Schmitz FJ , Petridou J , Astfalk N , Scheuring S , Köhrer K , Verhoef J , Fluit AC , Schwarz S . 2001. Structural alterations in the translational attenuator of constitutively expressed erm(A) genes in Staphylococcus aureus. Antimicrob Agents Chemother 45:1603–1604. doi:10.1128/AAC.45.5.1603-1604.2001 11372641 PMC90519

[26] Schmitz F-J , Petridou J , Jagusch H , Astfalk N , Scheuring S , Schwarz S . 2002. Molecular characterization of ketolide-resistant erm(A)-carrying Staphylococcus aureus isolates selected in vitro by telithromycin, ABT-773, quinupristin and clindamycin. J Antimicrob Chemother 49:611–617. doi:10.1093/jac/49.4.611 11909834

[27] Lampson BC , Parisi JT . 1986. Naturally occurring Staphylococcus epidermidis plasmid expressing constitutive macrolide-lincosamide-streptogramin B resistance contains a deleted attenuator. J Bacteriol 166:479–483. doi:10.1128/jb.166.2.479-483.1986 3084450 PMC214629

[28] Fines M , Gueudin M , Ramon A , Leclercq R . 2001. In vitro selection of resistance to clindamycin related to alterations in the attenuator of the erm(TR) gene of Streptococcus pyogenes UCN1 inducibly resistant to erythromycin. J Antimicrob Chemother 48:411–416. doi:10.1093/jac/48.3.411 11533008

[29] Rosato A , Vicarini H , Leclercq R . 1999. Inducible or constitutive expression of resistance in clinical isolates of streptococci and enterococci cross-resistant to erythromycin and lincomycin. J Antimicrob Chemother 43:559–562. doi:10.1093/jac/43.4.559 10350387

[30] Dubnau D . 1985. Induction of ermC requires translation of the leader peptide. EMBO J 4:533–537. doi:10.1002/j.1460-2075.1985.tb03661.x 4018035 PMC554218

[31] Weisblum B . 1995. Insights into erythromycin action from studies of its activity as inducer of resistance. Antimicrob Agents Chemother 39:797–805. doi:10.1128/AAC.39.4.797 7785974 PMC162632

[32] Werckenthin C , Schwarz S , Westh H . 1999. Structural alterations in the translational attenuator of constitutively expressed ermC genes. Antimicrob Agents Chemother 43:1681–1685. doi:10.1128/AAC.43.7.1681 10390222 PMC89343

[33] Schmitz F-J , Petridou J , Astfalk N , Köhrer K , Scheuring S , Schwarz S . 2002. Molecular analysis of constitutively expressed erm(C) genes selected in vitro by incubation in the presence of the noninducers quinupristin, telithromycin, or ABT-773. Microb Drug Resist 8:171–177. doi:10.1089/107662902760326878 12363005

[34] Treangen TJ , Maybank RA , Enke S , Friss MB , Diviak LF , Karaolis DKR , Koren S , Ondov B , Phillippy AM , Bergman NH , Rosovitz MJ . 2014. Complete genome sequence of the quality control strain Staphylococcus aureus subsp. aureus ATCC 25923. Genome Announc 2:e01110-14. doi:10.1128/genomeA.01110-14 25377701 PMC4223452

[35] Lim J-A , Kwon A-R , Kim S-K , Chong Y , Lee K , Choi E-C . 2002. Prevalence of resistance to macrolide, lincosamide and streptogramin antibiotics in Gram-positive cocci isolated in a Korean hospital. J Antimicrob Chemother 49:489–495. doi:10.1093/jac/49.3.489 11864949

[36] Spiliopoulou I , Petinaki E , Papandreou P , Dimitracopoulos G . 2004. Erm(C) is the predominant genetic determinant for the expression of resistance to macrolides among methicillin-resistant Staphylococcus aureus clinical isolates in Greece. J Antimicrob Chemother 53:814–817. doi:10.1093/jac/dkh197 15056638

[37] Barros EM , Ceotto H , Bastos MCF , Dos Santos KRN , Giambiagi-Demarval M . 2012. Staphylococcus haemolyticus as an important hospital pathogen and carrier of methicillin resistance genes. J Clin Microbiol 50:166–168. doi:10.1128/JCM.05563-11 21976766 PMC3256717

[38] Musser JM , Kapur V . 1992. Clonal analysis of methicillin-resistant Staphylococcus aureus strains from intercontinental sources: association of the MEC gene with divergent phylogenetic lineages implies dissemination by horizontal transfer and recombination. J Clin Microbiol 30:2058–2063. doi:10.1128/jcm.30.8.2058-2063.1992 1500513 PMC265442

[39] Archer GL , Thanassi JA , Niemeyer DM , Pucci MJ . 1996. Characterization of IS1272, an insertion sequence-like element from Staphylococcus haemolyticus. Antimicrob Agents Chemother 40:924–929. doi:10.1128/AAC.40.4.924 8849253 PMC163232

[40] Takeuchi F , Watanabe S , Baba T , Yuzawa H , Ito T , Morimoto Y , Kuroda M , Cui L , Takahashi M , Ankai A , Baba S , Fukui S , Lee JC , Hiramatsu K . 2005. Whole-genome sequencing of Staphylococcus haemolyticus uncovers the extreme plasticity of its genome and the evolution of human-colonizing staphylococcal species. J Bacteriol 187:7292–7308. doi:10.1128/JB.187.21.7292-7308.2005 16237012 PMC1272970

