# Supplementary figures and images for: Molecular basis and evolutionary cost of a novel macrolides/lincosamides resistance phenotype in Staphylococcus haemolyticus
Source: Microbiol Spectr. 2023 Sep 19;11(5):e00441-23. doi: 10.1128/spectrum.00441-23 (PMC10655708; doi:10.1128/spectrum.00441-23)

Fig. S1

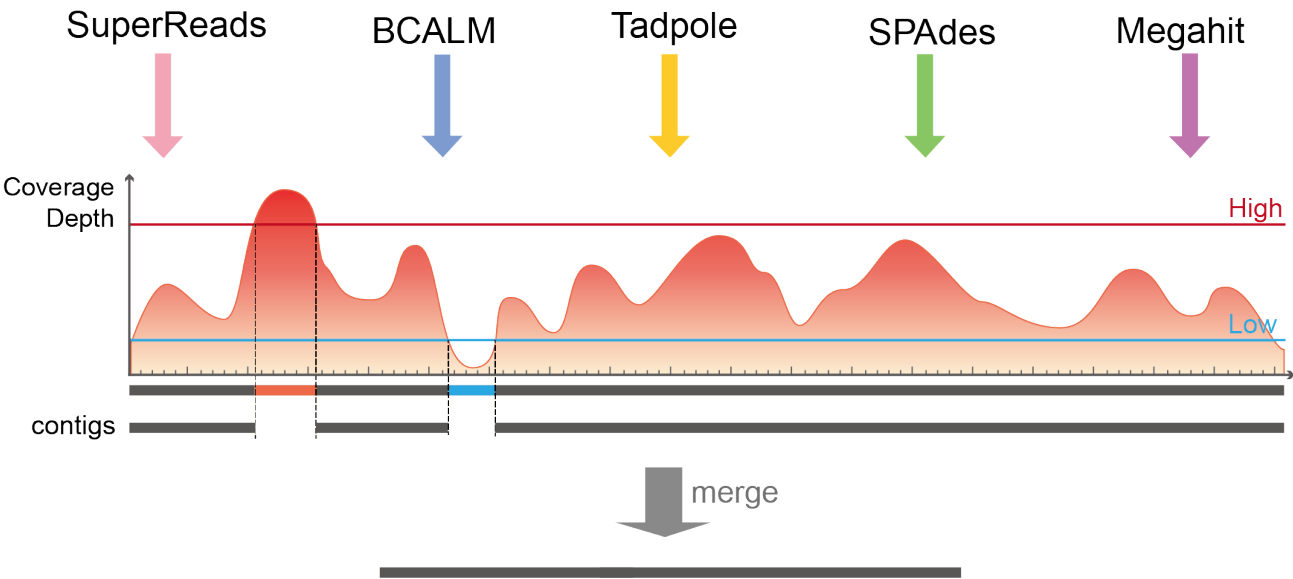

Fig. S2

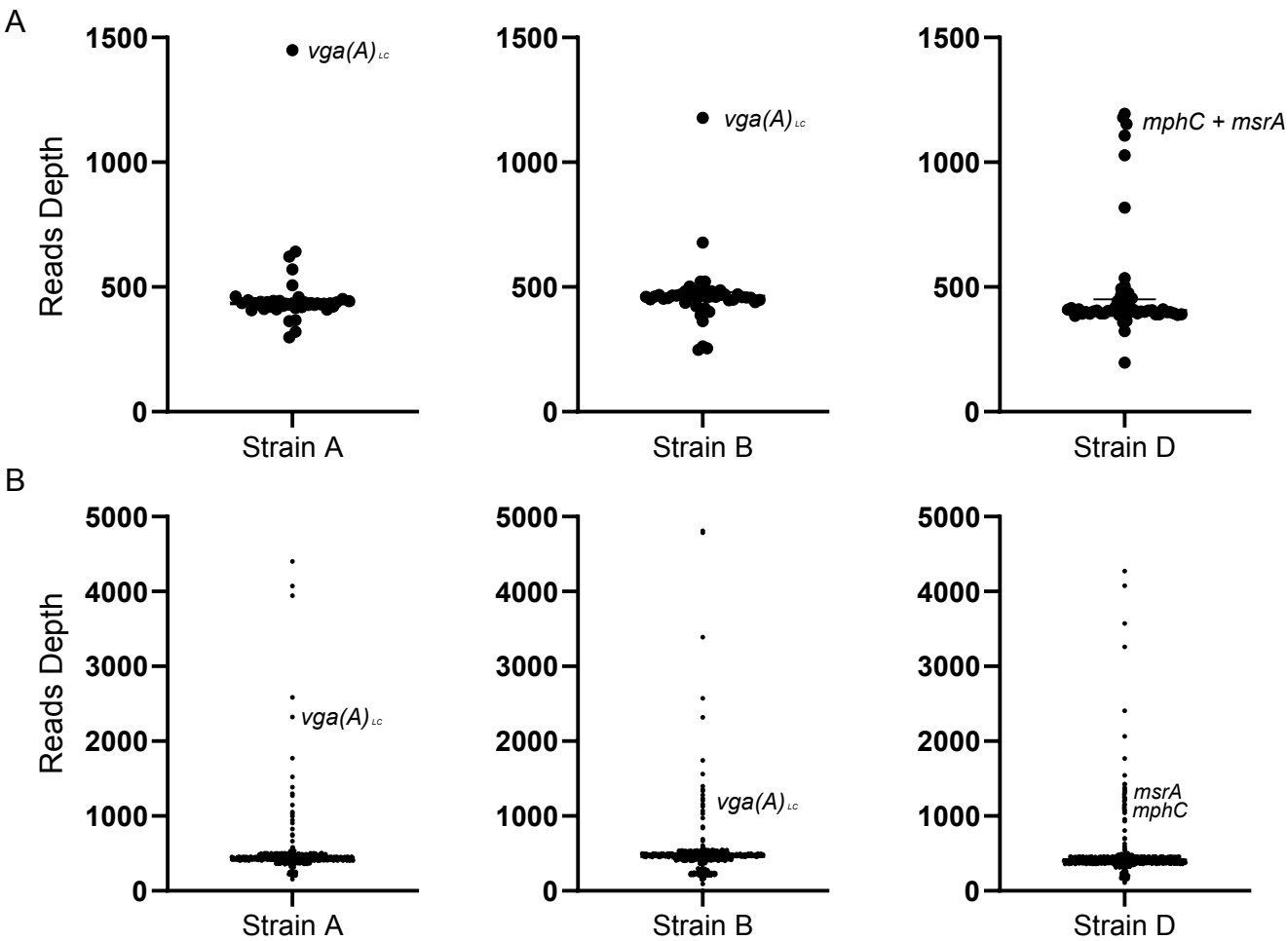

Supplement: Figures S1 and S2 — Schematic diagram of merge contigs in genome assembly process. [file spectrum.00441-23-s0001.pdf]

**Fig. S3**

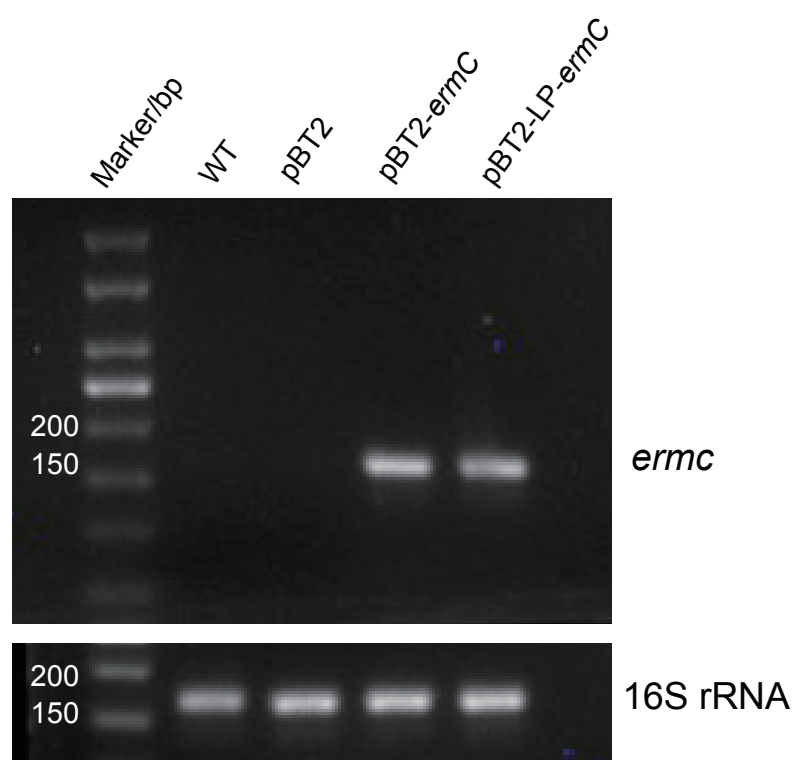

Supplement: Figure S3 — Identification of ermC expression in S. aureus ATCC25923 transformed with different plasmids. [file spectrum.00441-23-s0002.pdf]
