# Supplementary material for: Molecular basis and evolutionary cost of a novel macrolides/lincosamides resistance phenotype in Staphylococcus haemolyticus
Source: Microbiol Spectr. 2023 Sep 19;11(5):e00441-23. doi: 10.1128/spectrum.00441-23 (PMC10655708; doi:10.1128/spectrum.00441-23)
Supplement: Tables S1 and S2 — All potential resistance genes identified in S. haemolyticus strains ABCD; primers used in this study. [file spectrum.00441-23-s0003.pdf]

Table. S1

Gene	*S. haemolyticus* Strains A	B	C	D	Gene Description	Antibiotic Resistance
AAC(6')-Ie-APH(2'')-			■	■	aminoglycoside N-acetyltransferase AAC(6')-Ie/ aminoglycoside O-phosphotransferase APH(2'')-Ia	aminoglycoside
Ia APH(3')-IIIa		■	■		aminoglycoside phosphotransferase	aminoglycoside
catA8			■		chloramphenicol acetyltransferase	phenicol
dfrG			■		trimethoprim resistant dihydrofolate reductase dfr	diaminopyrimidine
ermC			■		Erm 23S ribosomal RNA methyltransferase	macrolide, lincosamide, streptogramin
mecA	■	■	■		beta-lactam-resistant peptidoglycan	penam
mphC			■	■	transpeptidase macrolide phosphotransferase	macrolide
msrA			■	■	ATP-binding cassette (ABC) transporter	macrolide, lincosamide, streptogramin, tetracycline, oxazolidinone, phenicol, pleuromutilin
PC1 beta-lactamase	■	■	■		blaZ beta-lactamase	penam
SAT-4		■	■		streptothricin acetyltransferase	nucleoside
tet(45)		■			tetracycline efflux MFS transporter	tetracycline
tet(K)				■	tetracycline efflux MFS transporter	tetracycline
vga(A)$_{LC}$	■	■			ATP-binding cassette (ABC) transporter	macrolide, lincosamide, streptogramin, tetracycline, oxazolidinone, phenicol, pleuromutilin

Table. S2

Primer Name	Primer Data
ermC-XbaI-F	GCTCTAGAGCAGTATAAATTTAACGATCAC
ermC-EcoRI-R	CGGAATTCCGATTCACAAAAAATAGGTACACG
In-fusion vector-F	GGTTATAATGAATCGTTAATAAGC
In-fusion vector-R	CTCTAGAGTCGACCTGCAG
In-fusion insert-F	CAGGTCGACTCTAGAGCAGTATAAATTTAACGATCACTCA
In-fusion insert-R	CGATTCATTATAACCACTTATTTTTTGTTTGGTTGATAAT
rt-*ermC*-F	CACAGTCAAAACTTTATTAC
rt-*ermC*-R	GGTCTATTTCAATGGCAGTTACG
rt-Staphylococcus 16S-F	CCATTGTAGCACGTGTGTAG
rt-Staphylococcus 16S-R	GAGATGTTGGGTTAAGTCCC
