# Supplementary material for: Molecular basis and evolutionary cost of a novel macrolides/lincosamides resistance phenotype in Staphylococcus haemolyticus
Source: Microbiol Spectr. 2023 Sep 19;11(5):e00441-23. doi: 10.1128/spectrum.00441-23 (PMC10655708; doi:10.1128/spectrum.00441-23)
Supplement: Supplemental material information — Legends for supplemental figures and tables. [file spectrum.00441-23-s0004.pdf]

Supplementary Information

Fig S1. Schematic diagram of merge contigs in genome assembly process. The red peak represented coverage depth, the pure red or light blue line represented high or low depth respectively. Assemblies of five tools (SuperReads, BCALM, Tadpole, SPAdes, Megahit) were screened according to the coverage depth of reads.

Fig S2. Reads depth of contigs (A) and annotated genes (B) from *S. haemolyticus* strain A, B and D. The resistance genes marked here were associated with erythromycin and clindamycin.

Fig S3. Identification of *ermC* expression in *S. aureus* ATCC25923 transformed with different plasmids. The upper showed the target fragment (*ermC*) and the lower showed the fragment of 16S rRNA internal reference.

Table S1. All potential resistance genes identified in *S. haemolyticus* strains ABCD.

Table S2. Primers used in this study.
